# Junctional ectopic tachycardia following repair of congenital heart defects-experience in multimodal management from a West African Centre

**Published:** 2012-06-01

**Authors:** Kow Entsua-Mensah, Ernest Aniteye, Lawrence Agyemang Sereboe, Mark Mawutor Tettey, Frank Edwin, Martin Tamatey, Ibrahim Delia, Kofi Bafoe Gyan

**Affiliations:** 1 National Cardiothoracic Centre, Korle-Bu Teaching Hospital, P. O. Box KB 846, Korle-Bu, Accra, Ghana; 2 Walter Sisulu Paediatric Cardiac Centre for Africa, Sunninghill Hospital, Cnr. Witkoppen & Nanyuki Rd.,Sunninghill Park, Sandton, Johannesburg; 3 Ahmadu Bello University Teaching Hospital, Zaria, Nigeria

**Keywords:** Junctional ectopic tachycardia, congenital, heart defects, amiodarone, magnesium sulphate, direct current shock, multimodal management

## Abstract

**Background:**

Postoperative junctional ectopic tachycardia (JET) is a rare and transient phenomenon occurring after repair of congenital heart defects. Report on this arrhythmia in the subregion is rare. We set out to determine the incidence of this arrhythmia and review the treatment and outcomes of treatment in our centre.

**Methods:**

Retrospective search of the records of all patients aged 18 years and below admitted into the intensive care unit (ICU) following repair or palliation of a congenital heart defect over 5 years, from January 1, 2006 to December 31, 2010. A review of clinical notes, operative records, anaesthetic charts, cardiopulmonary bypass (CPB) records, nursing observation charts, electrocardiograms (ECGs) and out-patient follow-up records was undertaken.

**Results:**

510 children under 18 years were enlisted. 7 cases of postoperative JET were recorded, (1.37%). 184 (36.1%) of these were performed under CPB. All JET cases were from cases done under CPB, 3.8%. Median age was 3 years and median weight 11.3kg. No patient was febrile at diagnosis. 4 patients had amiodarone administration, 5 had magnesium sulphate infusion, 2 patients had direct current shock (DCS) whilst 3 patients had all three therapeutic modalities. All patients had control of the arrhythmia with conversion to sinus rhythm and no recurrence.

**Conclusion:**

We report a JET incidence of 1.37% among children undergoing CPB for repair of congenital heart defects. We demonstrate the therapeutic effectiveness of amiodarone, magnesium sulphate infusions and DCS alone or in combination in the management of JET on various substrates with good outcome.

## Background

Postoperative JET is a rare cardiac arrhythmia characterized by atrio-ventricular dissociation, a high rate junctional escape rhythm and poor clinical tolerance [1, 2]. It occurs in 1-2% of all open-heart cases and more commonly after paediatric cardiac operations [3, 4, 5]. The incidence reaches up to 8-20% in certain operations [6]. The cause of JET is thought to be the result of an injury to the conduction system during a procedure and may be perpetuated by haemodynamic disturbances or postoperative electrolyte disturbances, namely hypomagnesemia [7]. The abnormal rhythm causes significant morbidity and mortality, secondary to decreased cardiac output [2, 8]. Pharmacologic and non-pharmacologic options may not result in reversal of the rhythm [2, 6].

Data on the incidence of this potentially hazardous arrhythmia is not available in our sub-region, where only a handful of centres are actively performing open-heart surgery. The aim of this study was to determine the incidence of JET among patients presenting for congenital heart repairs at the Cardiothoracic Centre in Accra and review the management and outcomes of management over the five year period.

## Methods

A retrospective review of admissions to our intensive care unit from January 1, 2006 to December 31, 2010 was carried out. We then reviewed the case notes, CPB records, nursing observation charts, ECGs and out-patient follow-up records of these patients. We noted the characteristics of these patients with respect to pre and postoperative diagnoses, procedure performed, time to diagnosis of JET and temperature at diagnosis, therapeutic modalities employed in the control of the arrhythmia, time to rate and rhythm control, the morbidity consequent upon the treatment of the arrhythmia and intensive care unit (ICU) length of stay.

### Inclusion criteria

All patients less than 18 years of age who had a procedure for repair or palliation of a congenital heart defect were enlisted into the study.

### Exclusion criteria

All patients aged more than 18 years and all patients less than 18 years but who had a procedure other than repair or palliation of a congenital heart defect were excluded from the study.

### Criteria for diagnosis of JET

Heart rate greater than 180 beats per minute with a narrow complex tachycardia and atrioventricular dissociation were our main criteria for diagnosis. Time of control of the tachycardia was defined as “the time the spontaneous pulse rate was first recorded at less than 180 beats per minute”[1].

### Analysis

Results are presented as median and range.

## Results

1,465 patients were admitted to the cardiothoracic ICU following various cardiothoracic and vascular procedures. Of these, 510, (34.1%) were admissions of children 18 years and below. Of the 510 paediatric surgical admissions 7, (1.37%) met criteria for the diagnosis of postoperative JET. 184 out of the 510 paediatric cases (36.1%) were performed under CPB. All JET cases arose from cases done under CPB, with an absolute risk of 3.8%.

Median age was 3 years, (1year 4 months-7 years). Median weight was 11.3kg, (7.0kg-21.0kg). We did not observe JET in any patient who had surgery without CPB.

4 patients had patch closure of ventricular septal defects, (VSD), 2 patients had repair of tetralogy of Fallot, (TOF), whilst 1 patient had repair of partial anomalous pulmonary venous connections, (PAPVC). No patient was febrile at diagnosis. Median temperature at diagnosis was 36.3°C, (35.1°C to 37.3°). Features of the patients who experienced JET are summarized in Table 1. Part of the ICU records for patient 1 could not be retrieved. The missing parameters were therefore excluded in the determination of the relevant median figures.

**Table 1:** Summary of patient characteristics, procedures performed management of JET, complications, outcomes and ICU stay, 2006 – 2010

Age (years)	Wt (kg)	Procedure	Temperature at diagnosis (^o^C)	Time from diagnosis to treatment	Amiodarone treatment regimen	Magnesium sulphate boluses	Direct current cardioversion (Joules)	Time until rate control	Adverse effects of JET treatment	ICU LOS (days)
7 years	19.9	Repair of TOF	Missing	Missing	Yes	**-**	-	Missing	Hypotension	44
1 year 4months	7.0	Repair of PAPVC and secundum ASD	37.2	10 hours	Yes	-	-	52 hours	Hypotension	10
2 years 7months	10.3	Patch closure of VSD	35.4	10 min	-	X 3	-	21 hours	Hypotension	6
4 years	11.3	Patch closure VSD, ligation of PDA	36.0	1 min	-	X 2	-	5 min	-	3
5 years 4 months	21.0	Repair of TOF	35.1	2 hours	-	X 1	-	2 hours 30 min	-	4
3 years	10.0	Patch closure of VSD	36.5	1 min	Yes	X 1	Intra op 10J x 6, Post op 20J x2	1 min (op)26 hr 30 min	Hypotension	4
2 years 10 months	14.9	Patch closure of VSD	37.3	1 min (op) 5 min(ICU)	Yes	X 1	Intra op 30J, 50J	3 min (op) 10 sec (ICU)	Hypotension	3

ICU: Intensive care unit, LOS: Length of stay, TOF: Tetralogy of Fallot, ARF: Acute renal failure, PAPVC: Partial anomalous pulmonary venous connections, ASD: Atrial septal defect, VSD: Ventricular septal defect, PDA: Patent ductus arteriosus, DCS: Direct current shock. X: number of times intervention was performed, Op: operation, Yr(s): year(s), Min: minutes

The incidence of JET for various surgical procedures have been previously published.[2] In our cohort, JET was noted in one out of two patients who had repair of anomalous pulmonary venous connections, 3.9% of those with repair of tetralogy of Fallot and anatomic variants(n = 51) and 4.5% of those who had ventricular septal defect repair (n = 89). Our observed incidences of JET for the particular procedures are summarized in Table 2.

**Table 2:** Procedures and estimated Junctional ectopic tachycardia frequencies, 2006-2010

	Total no. of cases	Cases with JET	Observed frequency %	Published frequency % [2]
Repair of VSD	89	4*	4.5	4 – 13
Repair of TOF	51	2	3.9	14 – 22
Repair of APVC	2	1	50	30

JET: Junctional ectopic tachycardia; VSD: ventricular septal defect, TOF: tetralogy of Fallot; APVC: anomalous pulmonary venous connections;

* One patient also had ligation of PDA

Median time from diagnosis to treatment was 10 minutes, (1 minute to 10 hours). There was no formal protocol for the management of JET over the study period. 4 patients had amiodarone infusion of 10mg/kg over 24 hours after a bolus of 5mg/kg over an hour. 5 had magnesium sulphate infusions of 50mg/kg/hour until serum magnesium levels were normalised. Maximum daily dose of magnesium sulphate did not exceed 2g in 24 hours. Two patients had DCS whilst 3 patients had multimodal management with all three therapeutic modalities described above. In those who had magnesium sulphate infusions, postoperative serum magnesium levels were 50-90% of the lower limit of normal. Magnesium sulfate infusion was continued until serum levels were in the normal range. All 4 patients receiving amiodarone therapy developed significant hypotension necessitating a step-up of the inotropic support. Other electrolyte deficiencies, mainly calcium and potassium, were controlled by boluses or continuous infusions respectively as necessary.

Routine general interventions instituted included sedation and analgesia with midazolam and morphine infusions, cooling with ice packs, paralysis and ventilation except the 5^th^ patient who was only sedated but not ventilated. Correction of biochemical abnormalities and reduction of inotropic support, where possible, was also carried out. Once the junctional rate was reduced, external cardiac pacing was instituted where necessary.

Median time to rate control was 2 hours 30 minutes, (5 minutes to 52 hours). Rate control was sustained in patients receiving amiodarone and magnesium infusions whilst those receiving DCS were not sustained and required repeated shocks. Median ICU stay was 4 days, (3 days to 44 days). Patient no.1 developed acute renal failure necessitating peritoneal dialysis. This was complicated by peritonitis. He also developed left empyema thoracis and sepsis with multi-organ dysfunction and required antibiotic treatment and chest drainage with an intercostal tube. Parenteral nutrition was instituted to complement nasogastric feeds. The peritoneal dialysis was continued despite the peritonitis. Renal function returned to normal and dialysis was discontinued after 21 days. He made complete recovery and was discharged to the ward after 44 days.

All patients who had amiodarone were continued on oral administration following discharge home. All had oral amiodarone therapy discontinued within 3 months and are doing well. None of the patients had a recurrence after the ICU event. Follow-up is complete, median 16 months (10 months to 56 months).

## Discussion

Postoperative JET, unlike the congenital variant, presents as a transient phenomenon immediately after surgery for congenital heart disease.[2, 3, 9, 10] It usually begins 6-72 hours following cardiopulmonary bypass surgery for repair of congenital heart lesions [5]. It occurs in 15 to 25% of infants following repair of TOF, in up to 12% of infants following repair of VSD, in 10% of infants following repair of AVSD, and in 30% of infants following repair of APVC.[2] The tachycardia may also occur in about 5% of infants after Fontan procedures, and can be encountered in infants after construction of a Blalock-Taussig shunt [2]. Our overall reported incidence of 1.37% compares with reports in literature [5].

The case-specific incidence we observed is also generally in agreement with the reported trends. Our observed incidence of JET in VSDs was 4.5%, close to the lower limit of the reported incidence. We employ the generally accepted technique of VSD patch closure with partial thickness bites on the right ventricular aspect of the VSD, away from the expected pathway of the conduction tissue. Our reported rate of 3.9% for repair of Fallot is also low compared to the incidence in literature. Here again we employ both myotomies and myectomies in the relief of the right ventricular outflow tract obstruction and closure of VSDs as detailed above. The incidence of JET in repaired partial anomalous pulmonary venous connections in our series was one out of two patients. It is a pointer to the behaviour of this anatomical substrate as it generates the highest reported percentage of JET following repair [2].

None of the cases performed for congenital heart defects without CPB in our cohort developed JET. Whilst our sample size is small, the particular susceptibility of patients undergoing CPB is noted in literature [2].

Injury to the conduction system is the proposed mechanism, incurred either by resection or excision of muscle bundles or relief of the right ventricular outflow tract through the right atrium commonly associated with a repair of tetralogy of Fallot [3, 7, 10]. Relief of right ventricular outflow tract obstruction appears to be more important in the causation of JET than does ventricular septal defect closure alone. Muscular resection seems to be more arrhythmogenic than is simple division [3, 7]. Autopsy reports of children with fatal JET have shown haemorrhagic tracks invading the AV bundle originating from sutures close to the conduction system [2, 7].

Postoperative JET is often described as “warming-up”, i.e., the onset is insidious and gradual as opposed to re-entrant tachycardias, which are sudden in onset. It may result in significant compromise to cardiac output due to loss of AV synchrony and ventricular filling in the heart already compromised following surgery and cardiopulmonary bypass.[5] A fall in blood pressure and cardiac output usually occur concomitantly,[10] and is commoner with higher heart rates.[5] It may be perpetuated by haemodynamic disturbances or postoperative electrolyte disturbances, namely hypomagnesemia [2, 5]. Serum magnesium levels were not routinely measured preoperatively in our patients, but postoperative levels were between 50 and 90% of the lower limit of normal.

**Figure 1:**
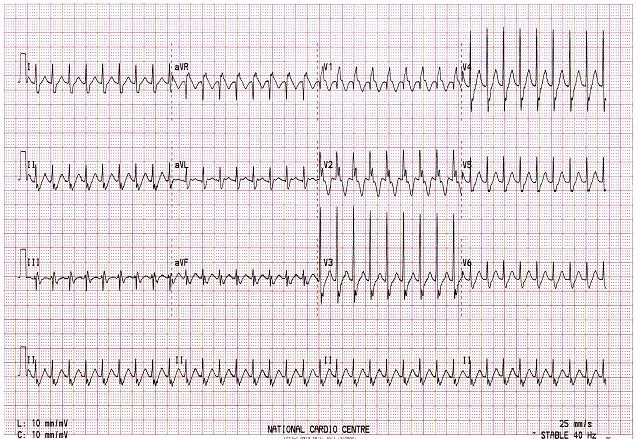
The QRS is usually a narrow complex and p-waves may be hidden, dissociated or retrograde

The diagnosis of JET usually is based on electrocardiographic evidence of a narrow complex tachycardia, heart rate ranging from 180 bpm to 300 beats/minute and regular with atrio-ventricular dissociation [7, 9]. The QRS is usually a narrow complex and p-waves may be hidden, dissociated or retrograde. This was confirmed in all our patients, (Figure 1). When there is retrograde conduction, adenosine infusion can rule out the diagnosis of atrioventricular reciprocating tachycardia, because of tachycardia cycle lengthening without termination of the arrhythmia. Diagnosis should be suspected in those anatomical substrates in which JET is common, i.e., TOF, VSD, AVSD and PAPVC. Atrial ECG is usually confirmatory in difficult cases. In comparing atrial ECG with surface ECG, atrio-ventricular dissociation can be clearly demonstrated [11]. None of our patients had atrial ECG performed since there was no doubt from the available standard 12-lead surface ECG recordings.

The electrophysiological mechanism of JET is thought to be abnormal automaticity within the His bundle consequent upon the injury [7]. It is usually unresponsive to overdrive pacing or DCS and may be resistant to unimodal therapy. Our 6^th^ and 7^th^ cases required multiple shocks, 8 and 2 shocks respectively in addition to administration of amiodarone and magnesium sulphate infusions to produce a sustained response.

Treatment of JET is indicated in infants with symptoms, reduced ventricular function or rapid heart rates. The management of infants with slow JET (less than 150 beats/min) without symptoms appear to be debated. However, the necessity to monitor accurately these asymptomatic patients is undoubted.

Historically, digoxin, propranolol and or amiodarone were used in the 1990s with variable success rates. Amiodarone, flecainide or propafenone, encainide or sotalol, alone or in combination have also been used. Currently, some studies single out amiodarone as having the highest response rate [2, 12]. It has been shown to be effective, alone, in decreasing the ventricular rate to less than 150 beats / minute in 50-70% of cases. It is credited with the most rapid control of the heart rate in post-operative JET as first line therapy, given either as a bolus followed by a continuous infusion, as was employed in our patients, or as repeated boluses.[9] Reported life threatening arrhythmias, however, suggest that intravenous amiodarone should be restricted to a setting where invasive monitoring and external cardiac pacing are available [2, 12]. Propafenone has been shown to be particularly effective in preventing or controlling the tachycardia only in patients with lower heart rates [4]. Other studies have employed the use of phenytoin, which was able to control ventricular rate but caused ataxia [7]. Ajmaline reduces ventricular rate but causes ventricular tachycardia after infusion [7]. Verapamil causes cardiovascular collapse.[7] In cases not responding to a single drug regimen, a combination of antiarrhythmic agents with different electrophysiological effects may control an otherwise untreatable congenital JET. Beta blockers like esmolol and sotalol have also been used effectively [7].

The pharmacological treatment of JET is characterised by a high rate of failure [7]. These therapies are also burdened by a high risk of toxicity as they have to be maintained at high dosages for long periods in young patients. In these cases the negative inotropic effects of antiarrhythmic drugs and the limitations in the use of sympathomimetic agents underscore this problem. Isoproterenol, dopamine, dobutamine and amrinone all increase the JET rate [7].

Magnesium supplementation in paediatric patients undergoing surgery for congenital heart defects has been known to have a protective effect from the complication of JET. This could be due to the stabilization of the membrane potential and the reduction of the automaticity, resulting in a low rate of development of the arrhythmia [2, 4, 13]. Magnesium sulphate supplementation after cessation of CPB may eliminate the occurrence of JET on admission into the intensive care unit [4, 5]. Better still, magnesium supplementation during the re-warming phase of cardiopulmonary bypass significantly reduces the incidence of JET, in a dose related manner [4, 13]. In our 4^th^ patient, we were able to abort the intraoperative pre-cardiopulmonary by-pass arrhythmias with magnesium sulphate boluses alone.

A staged management strategy is advised for JET resistant to unimodal pharmacologic therapy [9, 10]. This is demonstrated in our 6^th^ and 7^th^ cases which required use of amiodarone, magnesium sulphate and DCS.

General physiological measures such as avoidance of hyperthermia, sedation, minimizing catecholamines, active controlled cooling and correction of biochemical abnormalities or a combination of these have been found to be helpful [2, 5, 7, 10]. Therapeutic cooling may reduce the heart rate by 25-47 beats /min [14]. Hypothermia is known to decrease the automaticity of the heart, and has therefore been used alone to reduce heart rates in excess of 200 beats per minute to less than 180 beats per minute [15].

A definitive treatment for JET could be the removal of the arrhythmogenic area. Surgical His ablation has produced contrasting results. Radiofrequency, (RF) catheter ablation and cryoablation are able to control tachycardia and preserve normal AV conduction [7, 15].

## Conclusion

Postoperative JET is a potentially hazardous arrhythmia complicating palliation or repair of congenital heart defects. Certain anatomical substrates are at significant risk of developing this arrhythmia. In this report, we have demonstrated the therapeutic effectiveness of amiodarone, magnesium sulphate boluses and DCS alone or in combination in the management of this condition on various substrates with good outcome.
